# A dual role for α-synuclein in facilitation and depression of dopamine release from substantia nigra neurons in vivo

**DOI:** 10.1073/pnas.2013652117

**Published:** 2020-12-03

**Authors:** Mahalakshmi Somayaji, Stefano Cataldi, Se Joon Choi, Robert H. Edwards, Eugene V. Mosharov, David Sulzer

**Affiliations:** ^a^Department of Psychiatry, Columbia University, New York, NY 10032;; ^b^Division of Molecular Therapeutics, New York State Psychiatric Institute, New York, NY 10032;; ^c^Department of Physiology, University of California, San Francisco, CA 94518;; ^d^Department of Neurology, University of California, San Francisco, CA 94158;; ^e^Department of Neurology, Columbia University, New York, NY 10032;; ^f^Department of Pharmacology, Columbia University, New York, NY 10032

**Keywords:** dopamine, in vivo neurotransmission, alpha-Synuclein

## Abstract

α-Synuclein is expressed at high levels at presynaptic terminals, but defining its role in the regulation of neurotransmission under physiologically relevant conditions has proven elusive. We report that, in vivo, α-synuclein is responsible for the facilitation of dopamine release triggered by action potential bursts separated by short intervals (seconds) and a depression of release with longer intervals between bursts (minutes). These forms of presynaptic plasticity appear to be independent of the presence of β- and γ-synucleins or effects on presynaptic calcium and are consistent with a role for synucleins in the enhancement of synaptic vesicle fusion and turnover. These results indicate that the presynaptic effects of α-synuclein depend on specific patterns of neuronal activity.

## Significance

We report a long-sought& in vivo& physiological role for& α-synuclein (α-syn) in dopamine signaling. The results indicate that& α-syn is critical for activity-dependent dopamine plasticity, and that short repeated burst activity produces rapid presynaptic facilitation, while prolonged burst activity slowly depresses evoked dopamine release. We propose that the rapid facilitation is due to an enhanced fusion of synaptic vesicles at active zones during exocytosis while the depression is due to synaptic exhaustion. These results identify a& dynamic role of& α-syn, and are critical for defining& molecular mechanisms and therapeutic targets for various neurological disorders, where the firing properties of neurons are severely altered.

α-Synuclein (α-Syn) is an abundant and highly conserved cytosolic protein initially identified as a constituent of cholinergic presynaptic terminals that innervate the electric organ of the torpedo electric fish (1) and presynaptic inputs to cerebellar Purkinje cells (2). α-Syn has been reported to constitute 0.5 to 1% of the total protein in human and rat brain (3). Two closely related genes, β- and γ-synuclein (β-Syn and γ-Syn) are present in brain and peripheral organs (4). α-Syn was further identified as a major constituent of disease-related protein aggregates (5), including the Lewy body inclusions in Parkinson’s disease and other neurodegenerative disorders (6). Rare α-Syn mutant alleles (7) and genetic duplications and triplications (8) cause familial forms of Parkinson’s disease. Although the precise functions of synuclein isoforms remain unclear, they have been implicated in the modulation of synaptic vesicle fusion (9), while β-Syn has been suggested to inhibit α-Syn aggregation, and γ-Syn has been linked to multiple cancers and has been suggested to regulate signaling pathways and the cytoskeleton, and to act as a molecular chaperone (10).

Synucleins are amphipathic molecules that bind to acidic phospholipids on synaptic vesicles and other highly curved membranes (11). Following synaptic vesicle fusion, fluorescently tagged α-Syn dissociates from the vesicles to disperse from sites of exocytosis (12). To date, only modest effects on neurotransmitter release have been reported in studies of synuclein-deficient animals, consisting of a small increase in the time required for presynaptic recovery following a stimulus, and an increase in dopamine (DA) release from “triple knockout” (SynTKO) mice lacking α-, β-, and γ-Syn (9, 13–17).

It was recently shown that the synucleins act to enhance the rate of fusion pore dilation during secretory vesicle exocytosis (9). This effect was deduced from the slowed exocytosis of neuropeptides from large dense core vesicles in SynTKO mice. The neuropeptides are larger than classical small molecule neurotransmitters and require a longer time to diffuse from the vesicle lumen during rapid transient synaptic vesicle fusion events (∼50 µs for DA in synaptic vesicles) (18). It is unclear how synucleins might affect neurotransmitter release from small synaptic vesicles.

We conjectured that if synucleins promote vesicle pore dilation, they may selectively enhance neurotransmitter release during bouts of high neuronal activity. This might occur by enhancing the clearance of vesicle membrane from presynaptic active zones so that other synaptic vesicles might “refill” the active zone. A mechanism of this type would be particularly important at synapses that undergo bursts in the midst of ongoing tonic activity, such as from release sites on axons of ventral midbrain DA neurons where limited levels of presynaptic scaffolding proteins appear to constrain the number of active zones available for synaptic vesicle fusion (19, 20). The acute striatal slice preparation provides a useful system for studying DA release and reuptake, but lacks important attributes for differentiating the effects of axonal physiological stimulus patterns, as the DA cell bodies are absent and the axons do not receive physiologically relevant regulation from a variety of systems, including the ongoing activity of cortical and thalamic inputs, that are important in vivo (21).

To determine whether synucleins regulate the release of classical neurotransmitters from synaptic vesicles during physiologically relevant tonic and phasic activity, we stimulated the cell bodies of substantia nigra (SN) midbrain neurons and characterized evoked striatal DA release and DA axonal calcium transients in vivo under a state of light anesthesia. The results reveal that, in wild-type (WT) mice, α-Syn exerts a profound burst firing-dependent facilitation of DA release, consistent with a more rapid turnover of synaptic vesicle membrane and access of synaptic vesicles to presynaptic active zone fusion machinery, as well as a far slower stimulus-dependent longer-term depression. The faciliatory role for α-Syn identified here may be particularly important for synapses that undergo prolonged bouts of intermittent high activity, including those of monoaminergic neurons.

## Results

### Tonic and Bursting Activity of Ventral Midbrain Dopaminergic Neurons under Isoflurane Anesthesia.

During awake behavior, ventral midbrain DA neurons exhibit tonic firing that is interrupted by bursts of activity associated with environmental stimuli, including unexpected rewards and behaviorally salient cues, as reported in rodents (22) and nonhuman primates (23). In mice, the tonic pacemaking activity of DA neurons consists of action potentials at frequencies of 1 to 8 Hz that are dependent on the activities of L-type calcium channels and hyperpolarization and cyclic nucleotide-gated (HCN) channels (24) while bursting is controlled by a combination of excitatory and inhibitory synaptic inputs (25).

**Fig. 1.**
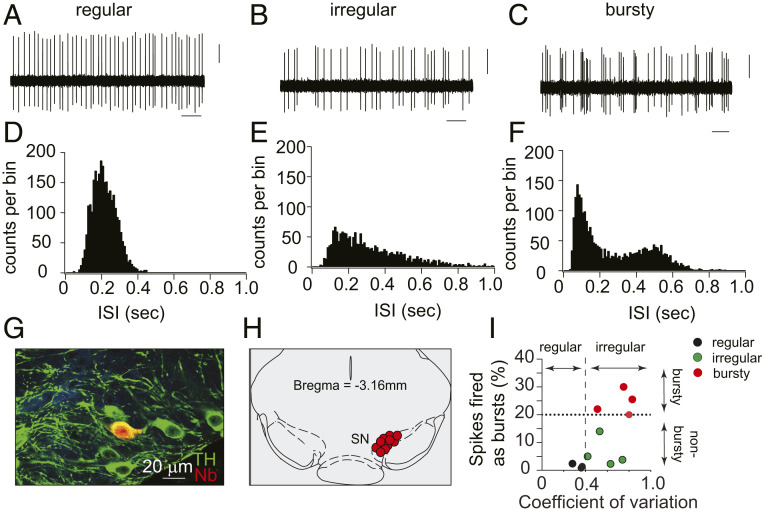
In vivo electrophysiological recordings from SN dopaminergic neurons in lightly anesthetized mice. (*A*–*C*) Representative extracellular in vivo recording of spontaneous firing activity of SN dopaminergic neurons, showing examples of regular (pacemaker, *A*), irregular (*B*), and burst firing (*C*) patterns. (Scale bars: 1 s and 5 mV.) (*D*–*F*) Representative histograms of interspike intrevals (ISIs) of individual regular (*D*), irregular (*E*), and bursty (*F*) neurons, demonstrating variations in firing patterns. (*G*) In vivo juxtacellular and immunocytochemical labels showing the neurochemical identity and anatomical localization of recorded dopaminergic neurons. Confocal laser scanning microscopic images of mouse brain tissue show neurons immunolabeled for TH (green) to identify dopaminergic neurons and neurobiotin (Nb, red) to visualize a neuron near the recording micropipette. (*H*) Anatomical mapping of all recorded WT DA neurons (*n* = 11) and their localization within the SN [coronal midbrain image adapted from Franklin and Paxinos’ *The Mouse Brain in Stereotaxic Coordinates*, Fourth Edition (58)]. (*I*) Scatter plot showing the coefficient of variation (mean/SD) and a fraction of spikes fired as bursts (SFB) of identified midbrain DA neurons. A dotted line at spikes fired as bursts (SFB) 20% represents the threshold for neurons classified as bursty together with respective autocorrelogram-based classifications. Note that ∼35% of the SN dopaminergic neurons exhibited burst firing under isoflurane anesthesia.

We recorded the spontaneous firing activity of SN midbrain neurons by extracellular in vivo recordings from WT mice during isoflurane anesthesia (26), from which mice recover within seconds following removal of the isoflurane. These neurons displayed firing properties typical of dopaminergic neurons, including their distinctive tonic, irregular, and phasic firing patterns (Fig. 1 *A*–*F*). We then performed juxtacellular labeling of the cells next to the recording electrodes, followed by post hoc immunolabeling for tyrosine hydroxylase (TH), which confirmed that the recorded cells were dopaminergic neurons of the SN pars compacta **(**Fig. 1 *G* and *H*).

To analyze the spontaneous in vivo activity of these neurons during the anesthesia, we quantified “burstiness” using a criterion defined by Grace and Bunney (27) where an interspike interval (ISI) of ≤80 ms defines the start of the burst, and ISI of ≥160 ms the end of the burst. Neurons were classified as “bursty” if the fraction of spikes fired as bursts (SFB) was greater than 20% of the total number of action potentials **(**Fig. 1*I*). The firing patterns of identified dopaminergic neurons were classified as regular or irregular based on autocorrelograms for each neuron (*Materials and Methods*).

In mice undergoing light (1%) isoflurane anesthesia, the majority of dorsal striatum-projecting dopaminergic SN neurons (7 of 11) fired tonically (nonbursty), and about 35% (4 of 11) displayed bursts interspersed by tonic activity. We then compared the firing rates of SN neurons of mice exposed to 1%, 3%, and 1% isoflurane in series. As expected, 3% isoflurane& slowed breathing more than 1%, but both levels showed identical tonic firing rates (*SI Appendix*, Fig. S1*D*) (1%, 4.9 ± 0.3; 3%, 4.8 ± 0.3; 1%, 4.5 ± 0.3, nonsignificant by one-way ANOVA). These tonic firing rates are similar to those previously reported in awake behaving rodents (*SI Appendix*, Table S1). While we cannot rule out a possibility that light isoflurane anesthesia may completely silence some dopaminergic neurons, this seems unlikely as cells that alternated between tonic activity and silence were not observed. Importantly, these firing patterns under light isoflurane anesthesia contrast with an absence of tonic activity reported in a fraction of neurons during deeper anesthesia with chloral hydrate (28).

We also examined whether isoflurane altered intrinsic firing properties of SN dopaminergic neurons by recording firing activity in acute midbrain slices using a cell-attached configuration. We found that the spontaneous neuronal firing rates were not different during exposure to 0% and 1% or 0% and 3% isoflurane in series (*SI Appendix*, Fig. S1*N*, control, 3.5 ± 0.3; 1% isoflurane, 3.6 ± 0.3; and *SI Appendix*, Fig. S1*Q*, not significant [ns], control, 3.1 ± 0.3; 3% isoflurane, 3.4 ± 0.5). We conclude that the anesthesia protocol used in our experiments has no effect on the rate of intrinsically generated pacemaking activity of SN pars compacta dopaminergic neurons.

### Characterization of Stimulus Parameters to Elicit DA Release.

Since the introduction of the carbon fiber microelectrode (29), amperometry and cyclic voltammetry have been used to measure evoked DA release and reuptake in anesthetized rodents (21). We used fast scan cyclic voltammetry (FSCV) to measure extracellular DA at subsecond temporal resolution.

**Fig. 2.**
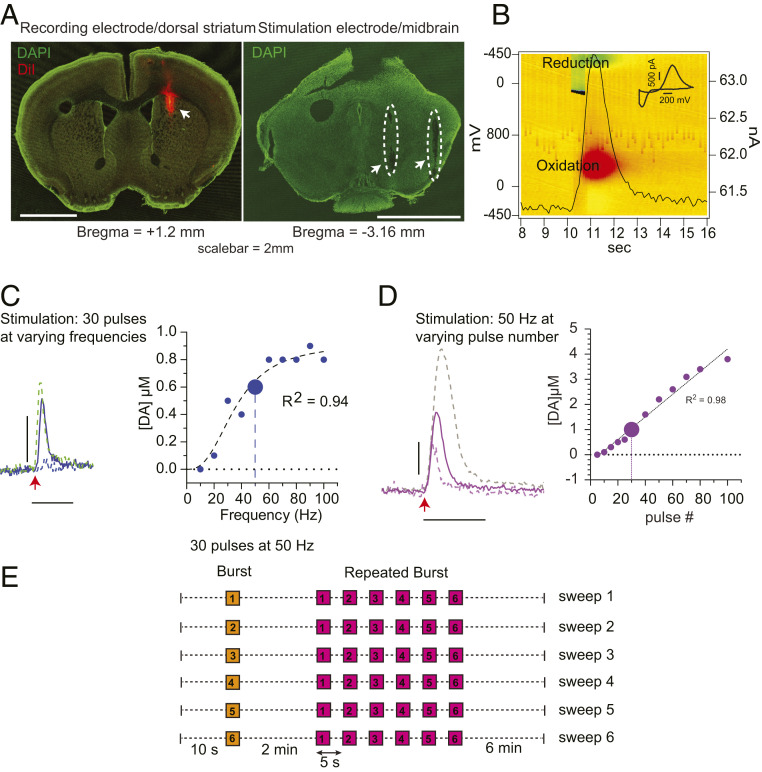
Protocol for the characterization of evoked DA release by FSCV in vivo. (*A*) Confocal laser scanning microscope images (5× objective) of coronal mouse brain sections indicating the location of recording electrode in the dorsal striatum (*Left*, green, DAPI; red, DiI staining, arrow) and the bipolar stimulating electrode in the ventral midbrain (*Right*, dashed outline and arrows) (Scale bar, 2 mm.) (*B*) Three-dimensional (3D) pseudocolor plot showing oxidation (red) and reduction (green) of DA. The time course of oxidation at 300 mV is superimposed as a black trace. (*Inset*) Voltammogram at the maximum of oxidation (11.2 s). (Scale bar: 200 mV and 500 pA.) (*C*, *Left*) Representative peaks of evoked DA release obtained by stimulating the dopaminergic cell bodies in the midbrain with a constant pulse number (30 pulses) at 20 Hz (dashed), 50 Hz (line), and 90 Hz (green, dashed) frequencies. The red arrow indicates the start of an electrical train of stimuli. (*C*, *Right*) Correlation between evoked DA release and stimulus frequency (blue, fit of the data with an allosteric sigmoidal curve *y = V*_max_*x^h/(*K*_*1/2*_*^h + x^h*), where *V*_*max*_ = 0.9 µM, *K*_*1/2*_ *=* 36 Hz, and *h* = 2.7; * = multiplication, ^ = caret/exponent symbol). (*D*, *Left*) Examples of evoked DA release following stimulation of midbrain dopaminergic neurons by 20 (dashed), 30 (line), and 60 (gray, dashed) pulses at a constant 50-Hz frequency. The red arrow indicates the start of the train of stimuli. (Scale bar: *y* axis, 500 nM DA; *x* axis, 5 s.) (*D*, *Right*) Correlation between evoked DA release and pulse number (magenta, linear regression *y* = 0.04x − 0.3). The larger circle represents parameters that provide a preferred dynamic range (30 pulses at 50 Hz) used in this study. (*E*) Stimulus protocol developed based on the results from *C* and *D*. Each sweep is comprised of a single stimulus train (orange, 30 pulses at 50 Hz) with a recovery period of 2 min, followed by a repeating burst stimulation (magenta), consisting of six stimulus trains (30 pulses at 50 Hz) every 5 s, with a recovery period of 6 min before the next sweep. The entire protocol consists of six consecutive sweeps. (*C* and *D*) Horizontal dotted line represents zero line, vertical dotted line represents the chosen parameters for the study.

To confirm the placement of the carbon fiber microelectrode, we coated it with a fluorescent lipophilic membrane dye, 1,1′-dioctadecyl-3,3,3′3′-tetramethyl-indocarbocyanine perchlorate (DiI). The bipolar stimulation electrode tracks in the ventral midbrain and the DiI-stained recording electrode track in the dorsal striatum were clearly observed in postfixed brain slices (Fig. 2*A*).

Burst firing by ventral midbrain DA neurons can be followed by a pause in tonic activity that is due to depolarization block. To determine whether our electrode placement reliably elicited this pause, we examined the response of individual SN neurons to antidromic stimuli applied to the regions of the dorsal striatum in which DA release is measured (*SI Appendix*, Fig. S2). While neuronal activity during the stimulus could not be measured due to a large electrical stimulus artifact, the stimuli elicited a pause in tonic firing by 8 of 9 individual SN neurons recorded, each of which lasted less than 5 s, after which the neurons resumed tonic firing. We conclude that 50-Hz stimuli reliably elicit burst firing by individual SN neurons.

Pioneering studies by Francois Gonon and coworkers demonstrated that, in the absence of reuptake blockers, stimuli that emulate the tonic activity of ventral midbrain DA neurons do not produce DA release in vivo that can be measured by electrochemistry (30), which typically has a limit of detection of ∼50 nM DA (21, 31). In contrast, burst firing of DA neurons at >10 Hz can drive DA levels to 1 µM or higher (32, 33) as a buildup of extracellular transmitter saturates the DA uptake transporter (DAT) (21, 34).

The oxidation profile and time course of DA released in the dorsal striatum evoked by the burst firing stimulus in the SN is shown as a color plot in Fig. 2*B*. The characteristic background-subtracted voltammogram at the maximum of the oxidation peak is consistent with the release of DA (Fig. 2 *B*, *Inset*). To study the dependence of striatal DA release on SN stimuli, we compared extracellular DA levels as a function of stimulus frequency at a constant pulse number (30 pulses) and as a function of the number of pulses at a constant stimulus frequency (50 Hz).

At 10-Hz stimuli, we did not resolve evoked DA release, consistent with the in vivo studies mentioned above (Fig. 2*C*) (30). Increasing the frequency from 20 to 60 Hz evoked a prominent DA signal while further increase in frequency did not change the amplitude of the DA peak (Fig. 2*C*). These results are interesting in light of reports in acute slice and in vivo demonstrating that SN dopaminergic neurons exhibit maximum firing rates between 15 and 40 Hz (*SI Appendix*, Table S1) due to voltage-dependent potassium currents that slow the firing rate (25, 35). We conclude that, while electrical stimuli in the ventral midbrain likely do not drive individual neurons to fire at rates of higher than 15 to 40 Hz, the summed activity of the population increased with stimulus frequency until a saturation was reached at about 60 Hz, similar to previous reports in vivo (36, 37).

We then examined the dependence of extracellular DA on the number of pulses at 50 Hz, which was linear between 5 and 80 pulses (Fig. 2*D*). This indicates that, within bursts, the same amount of DA was released per pulse and that DA release recovered to a stable level within 20 ms.

Based on these results, we chose 30 pulses at 50 Hz as a stimulus to elicit a burst firing response that evoked DA release in a linear range that is expected to be optimal for detecting neuromodulation. As shown from the antidromic stimulation responses, 6-s intervals between bursts are adequate for the recovery of tonic activity.

Based on these characterizations, we then designed a protocol for the characterization of activity-dependent DA plasticity (Fig. 2*E*), consisting of a “single burst” (Fig. 2*E*, orange box, a train of 30 pulses at 50 Hz) followed by a recovery period of 2 min and a train of “repeated bursts” (Fig. 2*E*, numbered 1 to 6, magenta boxes) stimuli. The latter was comprised of six “single bursts” repeated at 5-s intervals. A “single burst” followed by a “repeated burst” constitutes a “sweep” that was repeated six times with intersweep intervals of 6 min. This protocol provides information on both short-term and long-term effects on DA presynaptic plasticity in vivo.

### α-Synuclein–Dependent Deficits in Activity-Dependent Presynaptic Recovery at Longer (Minutes) Stimulation Intervals.

We compared responses to the single burst stimuli in three mouse lines: WT, “triple knockout” deficient for α, β, and γ synucleins (SynTKO), and a line in which only α-Syn was deleted (α-SynKO) (17).

**Fig. 3.**
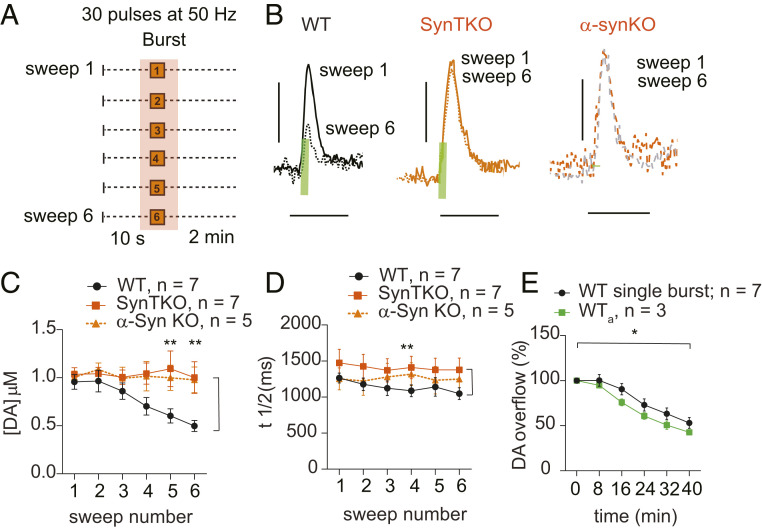
Synuclein-dependent decrease in evoked DA release during stimulation of midbrain neurons with single bursts and long (6-min) rest intervals. (*A*) Schematic of the stimulus paradigm. Shaded area highlights parts of the sweeps used for the analysis. (*B*) Evoked DA signal peaks following single burst stimulus (30 pulses at 50 Hz) from sweeps 1 and 6 showing the differences in DA release between WT (black; sweep 1, solid line; sweep 6, dotted line), synuclein triple knockout (SynTKO; orange; sweep 1, solid line; sweep 6, dotted line), and α−synuclein (α-SynKO sweep 1, orange dotted line; sweep 6, grey dotted line) mice. Green bars indicate electrical stimulation duration (0.6 s). (Scale bars: *y* axis, 500 nM DA; *x* axis, 5 s.) (*C*) Dopamine release decreases across sweeps in WT (black; one-way ANOVA within genotype: F_5,36_ = 5.2, *P* = 0.001; Tukey’s multiple comparison: sweep 1 vs. sweep 6, *P* = 0.006) but not in SynTKO (orange; one-way ANOVA within genotype: F_5,36_ = 0.073, *P* = 0.99, not significant by Tukey’s post hoc) and α-SynKO (orange-broken line; one-way ANOVA within genotype: F_5,24_ = 0.11, *P* = 0.99, not significant by Tukey’s post hoc) mice, resulting in significant difference between genotypes (two-way ANOVA between genotypes: F_2,96_ = 12.4, *P* < 0.0001; Tukey’s multiple comparison test showed significance between WT and SynTKO in bursts 5 and 6 (*P* = 0.003, 0.002 respectively), as well as WT and α-SynKO in bursts 5 and 6 (0.04, 0.009 respectively) (WT, n =7, SynTKO, n = 7, α-SynKO, n = 5). (*D*) The t_1/2_ did not change across sweeps in any of the genotypes. There was a shorter t_1/2_ in WT than in SynTKO, but not α-SynKO (two-way ANOVA: WT vs. SynTKO: F_1,72_ = 11.6, *P* = 0.001; WT vs. α-SynKO: F_1,60_ = 2.3, *P* = 0.12; WT, n = 7; SynTKO, n = 7; α-SynKO, n = 5). (*E*) Dopamine release decreased similarly in WT mice stimulated either with single burst within a sweep (black, same data as *C*) or single bursts only at 2-min intervals (green; one-way ANOVA within genotype: F_5,12_ = 37, *P* < 0.0001; Tukey’s multiple comparison: sweep 1 vs. sweep 6, *P* < 0.0001) (two-way ANOVA between genotypes: F_1,48_ = 5.8, *P* = 0.02. Bonferroni’s multiple comparison test did not show significance, n: WT, n = 7, WT_a_, n = 3). * = *P* < 0.05, ** = *P* < 0.005.

In WT mice, analysis of DA release evoked by single bursts (Fig. 3*A*) over the six sweeps showed a sweep number-dependent decrease to ∼50% of initial levels (Fig. 3 *B* and *C*, see figure legends for statistical details). This burst-firing–dependent decrease in DA release was not dependent on trains of bursts, as it also occurred when only single bursts (WT_a_) were applied (Fig. 3*E*). In contrast to WT, SynTKO and α-SynKO mice showed no decrease in evoked DA release (Fig. 3 *B* and *C*).

To investigate a possible role for synucleins in the regulation of DAT activity that could contribute to the stimulus-dependent changes in extracellular DA (38), we analyzed the reuptake kinetics by examining the peak half-width (t_1/2_ ) of the DA peaks. There was no change in t_1/2_ values within the sweeps in any of the genotypes, suggesting that DA reuptake was not altered during the course of the experiment (21, 34). We, however, noted a slightly longer t_1/2_ in the SynTKO than in WT (Fig. 3*D*).

These results indicate that α-Syn expression can cause an activity-dependent depression of DA signaling in vivo. This is consistent with previous reports both in vivo (15) and in vitro (14, 17) that synuclein expression slows the recovery of evoked DA release.

### α-Synuclein–Mediated Facilitation of DA Release at Short (Seconds) Stimulation Intervals.

**Fig. 4.**
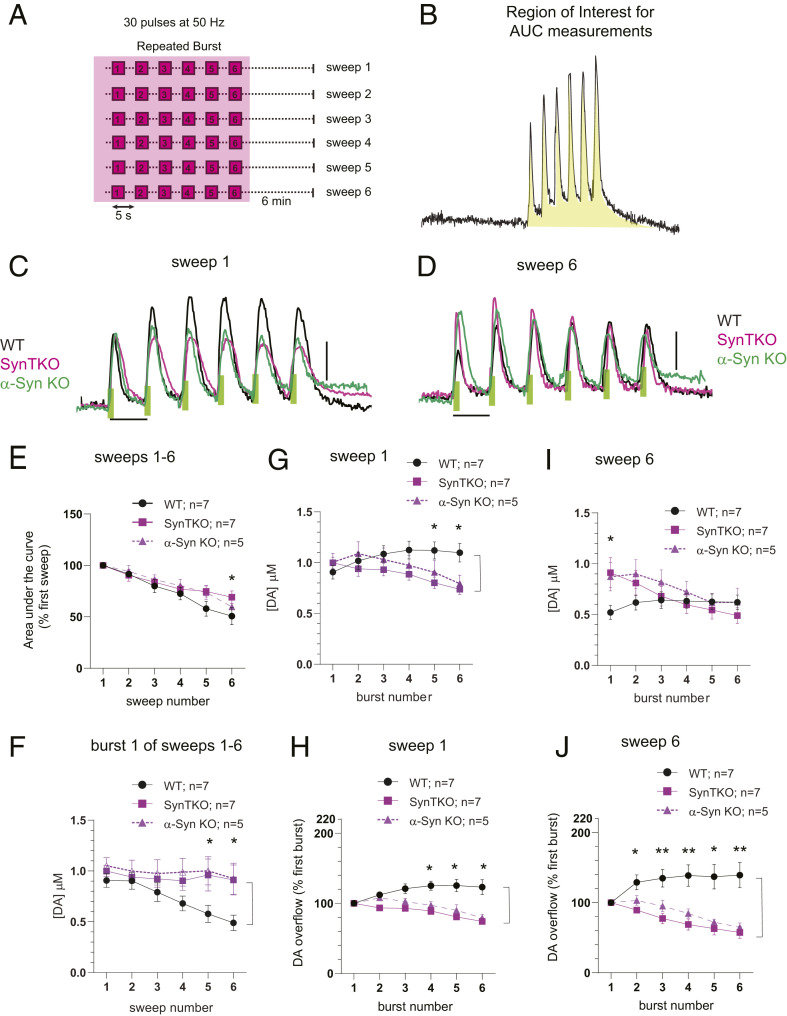
Synuclein-dependent facilitation of DA release during short-interval (5-s) burst stimuli. (*A* and *B*) Regions of the stimulation paradigm used for data analysis (shaded area). (*C* and *D*) Representative recordings of evoked DA release resulting from repeated burst stimulations during sweep 1 (*C*) and sweep 6 (*D*). WT (black) mice demonstrate short-term intraburst potentiation, while SynTKO (magenta) and α-SynKO (green) mice show short-term depression in vivo. Green bars indicate the duration (0.6 s) of electrical stimulation (Scale bar: *y* axis, 500 nM DA; *x* axis, 5 s.) (*E*) Depression of overall DA release during the entire train of repeated bursts duration measured as total AUC (as shown on *B*) across sweeps 1 to 6. Similar to single-burst data (Fig. 3*C*), depression of DA release was observed in WT mice (one-way ANOVA within genotype: F_5,36_ = 12.7, *P* < 0.0001; Tukey’s multiple comparison: sweep1 vs. sweep 6, *P* < 0.0001; *n* = 7) but not in SynTKO (one-way ANOVA within genotype: F_5,42_ = 0.8, *P* = 0.6; Tukey’s multiple comparison: not significant; *n* = 7) or α-SynKO (one-way ANOVA within genotype: F_5,24_ = 0.8, *P* = 0.6: Tukey multiple comparison: not significant; *n* = 5) animals. Two-way ANOVA between genotypes: F_2,96_ = 3.4, *P* = 0.04. Tukey’s multiple comparison: *P* = 0.04 between sweep 6 in WT and SynTKO. (*F*) Dopamine release in the first burst of repeated burst protocol decreases across sweeps in WT (one-way ANOVA within genotype: F_5,30_ = 21.9, *P* < 0.0001; *n* = 7) but not in SynTKO (one-way ANOVA within genotype: F_5,30_ = 0.29, *P* = 0.9; *n* = 7) and α-SynKO (one-way ANOVA within genotype: F_5,30_ = 0.41, *P* = 0.8 ; *n* = 5) mice. Two-way ANOVA between genotypes: F_2,96_ = 11.6, *P* < 0.0001; Tukey’s multiple comparison test: significance between WT and SynTKO in burst numbers 5 and 6 (*P* = 0.02, 0.007 respectively) and WT and α-SynKO between burst numbers 5 and 6 (*P* = 0.02, 0.01 respectively). (*G*) Facilitation of DA release during sweep 1 in WT (one-way ANOVA within genotype: F_5,30_ = 4.8, *P* = 0.0024; *n* = 7) and depression of DA release in SynTKO (one-way ANOVA within genotype: F_5,30_ = 13.0, *P* < 0.0001; *n* = 7) and α-SynKO (one-way ANOVA within genotype: F_5,20_ = 9.5, *P* < 0.0001 ; *n* = 5) animals during repeated trains of burst. Two-way ANOVA between genotypes: F_2,96_ = 7.9, *P* = 0.0006; Tukey’s multiple comparison test showed significance between WT and SynTKO in burst numbers 5 and 6 (*P* = 0.01, 0.004 respectively) and WT and α-SynKO between burst number 6 (*P* = 0.03). (*H*) Same data as *G* normalized to the first peak of each sweep. Two-way ANOVA between genotypes: F_2,96_ = 49.8, *P* < 0.0001; Tukey’s multiple comparison test showed significance between WT and SynTKO in burst numbers 2 to 6 (*P* = 0.04, 0.0008, <0.0001, <0.0001, <0.0001, respectively) and WT and α-SynKO between burst numbers 4 to 6 (*P* = 0.003, 0.0001, <0.0001, respectively). (*I*) Facilitation of DA release during sweep 6 in WT (one-way ANOVA within genotype: F_5,30_ = 2.9, *P* = 0.028) and depression of DA release in SynTKO (one-way ANOVA within genotype: F_5,30_ = 8.2, *P* < 0.0001; *n* = 7) and α-SynKO (one-way ANOVA within genotype: F_5,20_ = 6.1, *P* = 0.0013 ; *n* = 5) animals during repeated trains of burst. Two-way ANOVA between genotypes: F_2,96_ = 3.6, *P* = 0.03; Tukey’s multiple comparison test showed significance between WT and SynTKO in burst 1 (*P* = 0.006) and WT and α-SynKO between burst 1 (*P* = 0.03). (*J*) Same data as *I* normalized to the first peak of each sweep. Two-way ANOVA between genotypes: F_2,96_ = 49.8, *P* < 0.0001; Tukey’s multiple comparison test showed significance between WT and SynTKO in burst numbers 3 to 6 (*P* = 0.01, 0.0002, <0.0001, <0.0001, <0.0001, respectively) and WT and α-SynKO between burst numbers 4 to 6 (*P* = 0.03, 0.002, 0.0001, <0.0001, respectively). Data points represent mean ± SEM. * = *P* < 0.05, ** = *P* < 0.005.

We then compared DA release kinetics during repeated bursts of stimuli (Fig. 4*A*). Consistent with the response to single stimulus bursts (Fig. 3*C*), the overall amount of DA released during each sweep, measured as the area under the curve (AUC) (Fig. 4*B*, shaded area), decreased with successive sweeps in WT mice, but not in SynTKO and α-SynKO mice (Fig. 4*E*). Similarly, WT mice exhibited a progressive decrease in DA release elicited from the first burst within the repeated bursts train whereas release from SynTKO and α-SynKO mice was stable (Fig. 4*F*).

Remarkably, within each train of repeated bursts, the WT mice displayed a facilitation of evoked DA release whereas both SynTKO and α-SynKO mice showed a depression of release (Fig. 4 *G* and *I*). These responses were different between the genotypes in sweep 1 (Fig. 4 *C*, *G*, and *H*) and further increased in later sweeps (Fig. 4 *D*, *I*, and *J*).

As there are reports of differing effects of synuclein expression at older ages (39, 40), we also examined DA release kinetics in 10- to 13-mo-old mice (*SI Appendix*, Fig. S3). Similar to the younger 5- to 8-mo-old mice, older WT animals showed a facilitation and the mutants a depression of evoked release within the trains of bursts, indicating that these effects also occur in aged animals.

These data demonstrate a role for synucleins in facilitating neurotransmitter release in vivo and that this occurs during bouts of high presynaptic activity.

### Synuclein-Dependent DA Plasticity In Vivo Is Not due to Residual Presynaptic Calcium.

The classical model of presynaptic facilitation is that it results from an increased accumulation of residual calcium during closely-spaced repetitive stimuli (41). As SynTKO and α-SynKO mice displayed similar evoked DA release properties, we used the α-SynKO line to examine whether this mechanism might explain α-Syn–dependent presynaptic facilitation. We compared evoked calcium transients from DA axons in WT and α-SynKO mice by recording GCaMP6f signals by fiber photometry in the dorsal striatum, using the same coordinates and stimulus protocols detailed above (Fig. 2*E*) for electrophysiology and FSCV.

**Fig. 5.**
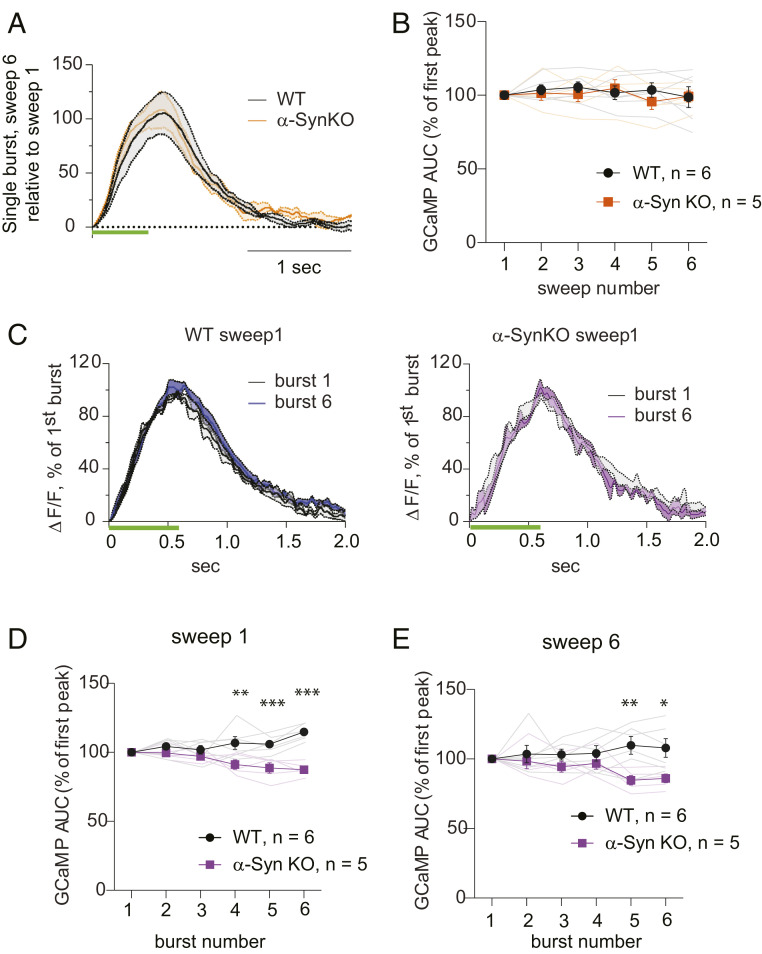
Calcium signals in response to burst stimuli in vivo. (*A*) Changes in evoked GCaMP6f signal transients during single burst stimulation in WT (black) and α-SynKO (orange) mice represented as averaged sweep 6 fluorescence relative to sweep 1 signal. No change in the amplitude or t_1/2_ was detected. (*B*) No change in the mean AUC of GCaMP responses at any sweep number was detected between the WT (black) and α-SynKO (orange) lines (two-way ANOVA between genotypes: F_1,54_ = 0.5, *P* = 0.5; Bonferroni’s multiple comparison test did not show significance; WT, n = 6; α-SynKO, n = 5). Faint traces show recordings from individual mice, while traces in bold represent average ± SEM. (*C*) Changes in GCaMP6f transients in sweep 1 represented as averaged signals at burst 6 normalized to burst 1 in WT and α-SynKO mice (ΔF/F, normalized GCaMP6f fluorescence). While the amplitude of evoked Ca^2+^ signal remained the same between WT (black) and α-SynKO (magenta) mice, there was a small increase in the t_1/2_ of the sixth GCaMP6f transient in WT neurons. Green bars indicate stimulus duration. (*D* and *E*) AUC of GCaMP transients in sweep 1 (*D*) and in sweep 6 (*E*) was significantly higher in WT than α-SynKO animals. Sweep1, two-way ANOVA between genotypes: F_1,54_ = 51.5, *P* < 0.0001; Tukey’s multiple comparison test showed significance between WT and α-SynKO in burst numbers 4 to 6 (*P* = 0.001, 0.0004, <0.0001, respectively). Sweep 6, two-way ANOVA between genotypes: F_1,54_ = 16.7, *P* = 0.0001; Tukey’s multiple comparison test showed significance in bursts 5 and 6 (*P* = 0.003, 0.01). WT, n = 6; α-SynKO, n = 5. The error bars represent SEM. * = *P* < 0.05, ** = *P* < 0.005, *** = *P* < 0.0005.

The AUC of the GCaMP signal depended linearly on the number of applied stimuli, while signals returned to baseline in <2 s after bursts (Fig. 5 and *SI Appendix*, Fig. S4). The averaged calcium transients evoked by single bursts of stimuli identical to those in Fig. 3*A* are shown in Fig. 5*A*. No difference in GCaMP signal response was observed between WT and α-SynKO mice (Fig. 5*B*).

The responses of GCaMP6f signals to train burst stimuli as in Fig. 4*A* are shown in Fig. 5 *C*–*E*. We observed a larger signal in the later bursts within sweeps in WT than α-SynKO animals, as seen from a small increase in t_1/2_ of the GCaMP peak. There was, however, no significant increase in signal in WT, particularly by sweep 6, where facilitation of DA release within bursts was observed, and we observed no buildup of GCaMP signal due to residual calcium. While the interpretation of the GCaMP6f signals has technical limitations (*Discussion* and *SI Appendix*, Fig. S4), differences in the accumulation of residual calcium appear unlikely to underlie the differences in DA release between WT and α-SynKO lines.

## Discussion

We examined the effects of synuclein expression on evoked DA release, reuptake, and axonal calcium transients in the dorsal striatum of mice lightly anesthetized with isoflurane. Our experimental conditions were designed to emulate the normal physiological patterns of ventral midbrain DA neuron activity during awake behavior, in which bursts are superimposed over pacemaking (tonic) activity. Our results indicate that α-Syn is involved in regulating both short-term and long-term DA plasticity in vivo. As the effects of α-SynKO and SynTKO on DA release in vivo were similar, α-Syn is apparently responsible for both the rapid facilitation and a slow depression of evoked DA release, and β-Syn and γ-Syn do not compensate for the loss of α-Syn at these synapses. This does not rule out a role for other synucleins in other synapses (9) or for dopaminergic synapses under other stimulus protocols.

While our experiments focus on the effects of synuclein expression on striatal DA neurotransmission, α-Syn expression is also reported to regulate the handling of synaptic vesicles at other synapses, including in hippocampal cultures and slices (9, 42, 43), and synuclein protein is found in nonneuronal cell types, such as glia and oligodendrocytes, and nonnervous system tissue, including kidney and red blood cells (44). As with all protocols that use electrical stimulus in the central nervous system, it is important to note that many cell types are activated, and electrical stimulation of the midbrain stimulates nondopaminergic cells (45). It is thus difficult to rule out circuit effects, particularly in vivo. For example, α-Syn is reported to affect synaptic plasticity via postsynaptic *N*-methyl-d-aspartate (NMDA) receptors in striatal cholinergic and medium spiny neurons, and these neurons also regulate striatal DA release (46–49).

The α-Syn–dependent facilitation of DA release is reminiscent of the classical presynaptic paired pulse facilitation extensively characterized at the neuromuscular junction and glutamatergic pyramidal synapses. These forms of facilitation, which are characterized at far shorter stimulus intervals, are thought to originate from low initial release probability (41) and a buildup of residual presynaptic calcium that enables the fusion of additional synaptic vesicles. We observed a slightly larger increase in GCaMP6f signal during trains of repeated bursts in WT than α-SynKO, but no evidence for a buildup of residual calcium between successive sweeps. It thus appears unlikely that α-Syn–dependent facilitation results from this classical mechanism of presynaptic facilitation. A note of caution is warranted, however, as the recording techniques do not indicate calcium changes within axonal compartments or may lack the sensitivity or temporal resolution to identify differences between the genotypes. Further experimentation and technology development will be needed to determine more subtle or local synuclein-dependent changes in dopaminergic axon calcium responses, if any.

**Fig. 6.**
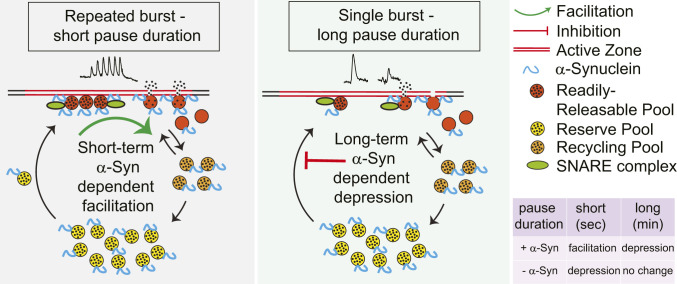
Model of α-Syn effects on DA release in vivo. In this model, the amount of neurotransmitter release is rate-limited by the pool of synaptic vesicle/active zone complexes competent for fusion upon stimulation-dependent increase in calcium (the readily releasable pool [RRP], red). During the short intervals between repeated bursts (seconds; *Left*), α-Syn enhances the dilation and collapse of synaptic vesicles, which in turn enhances the recovery of the active zones. Faster replenishment of the RRP and recycling pool (RP) allows a larger number of synaptic vesicles to fuse in response to subsequent stimuli. In the absence of synuclein, active zones remain occupied for longer durations, and the replenishment of the RRP and RP is slower, resulting in presynaptic depression. With substantially longer intervals between bursts (minutes; *Right*), the difference due to the rapid effects on synaptic vesicle dilation and collapse are negligible, revealing an inhibitory effect of α-Syn on a slower rate of transfer of vesicles from a reserve pool (yellow) to the RRP, leading to stimulation-dependent synaptic depression that is not observed in the absence of α-Syn. Black points represent the dopamine molecules (filled vesicle = circle with black points, empty vesicle = circle with no ponts).

An alternate mechanism for α-Syn–dependent presynaptic facilitation is suggested by a role for synucleins in facilitating secretory vesicle exocytosis by enhancing the rate of dilation and collapse of the vesicle membrane during fusion at active zones (9, 13). Multiple studies indicate that activity-dependent increase of presynaptic calcium promotes refilling of competent synaptic vesicle/active zone complexes (50, 51). A synuclein-dependent enhancement of this refilling rate initiated during burst firing-induced calcium entry could enable facilitation by a more rapid replacement of synaptic vesicles/active zone complexes (Fig. 6).

While the α-Syn–dependent facilitation of DA release is consistent with an increase of competent synaptic vesicle/active zone complexes, there are alternative explanations, including effects on SNARE proteins that enhance docking or fusion (52–54), enhancement of synaptic vesicle endocytosis (55), effects on the microtubule cytoskeleton (56), second messenger systems (43), and synaptic vesicle recycling. There is, moreover, evidence from amperometric recordings that most DA synaptic vesicle release events may occur via a partial release of synaptic vesicle content (18), and, if so, longer pore open times in the absence of synuclein expression could also enhance DA release. Unfortunately, quantal DA release events are too small to be measured in vivo with available technology.

As α-Syn expression in our experiments was responsible for a facilitation of DA release within bursts while α-SynKO and TKO exhibited a depression, α-SynKOs can exhibit a twofold relative facilitation. We note that α-Syn- and activity-dependent presynaptic facilitation of DA release was not reported in a prior in vivo study (15). This may be due to differences in the electrochemical detection technique (amperometry), site of electrical stimulation (medial forebrain bundle), stimulus paradigm (longer stimulation with less recovery time), or method of anesthesia (chloral hydrate, which can lead to silencing of both tonic and phasic activity by neurons) (28).

Consistent with some prior reports (14, 15), but not others (16), we found that DA release evoked by single bursts was initially similar in the dorsal striatum of WT, SynTKO, and α-SynKO mice but decreased only in WT in response to successive bursts separated by minutes-long pauses. The finding that synucleins can depress vesicle fusion in a calcium-independent manner appears consistent with our previous studies of the effects of α-Syn overexpression on quantal catecholamine release in chromaffin cells (57). In vivo, the slow depression in WT became apparent at >20 min, with the sixth burst releasing ∼50% of initial levels. Surprisingly, this slow depression was not enhanced by increasing the number of stimuli during this period and does not appear to be due to tissue damage as it was absent in the KO lines (Fig. 3 *C* and *E*).

The slow depression could be due to phosphorylated, aggregated, or multimeric synucleins that form following activity-dependent dissociation from synaptic vesicle membrane (12) and inhibit the mobility of synaptic vesicles that arrive over the course of minutes. These vesicles may be trafficked from “silent synapses” (19) where the release of DA from en passant sites on striatal dopaminergic axons appears to be limited by the presence of active zone scaffolding proteins (20) and/or SNARE proteins, vesicle transport, or other steps required for synaptic vesicle fusion (53, 54).

### Summary of Effects of Synuclein Expression on DA Release In Vivo.

We find that the tonic firing of SN DA neurons is retained during light anesthesia that does not alter tonic firing rates or evoked DA release (*SI Appendix*, Table S1 and Fig. S1) and that the levels of extracellular DA during tonic activity are below the detection limits of cyclic voltammetry (<50 nM). As tonic firing is far slower than the time required for presynaptic recovery within bursts (∼250 ms vs. < 10 ms), synuclein expression is expected to exert little effect on synaptic vesicle/active zone complexes and DA neurotransmission during tonic activity.

When a single burst exceeds five pulses at 50 Hz, DA reuptake is saturated, and extracellular DA is linearly dependent on the number of pulses. Bursts that occur at relatively long (minutes) intervals eventually engage an α-Syn–dependent slow depression of subsequent DA release, suggesting a decreased number of synaptic vesicle/active zone complexes. In contrast, during closely spaced repetitive bursts, there is an α-Syn–dependent facilitation of evoked DA release, consistent with an enhancement of competent synaptic vesicle/active zone complexes. The rapid facilitation can occur in tandem with the ongoing slow depression.

In conclusion, we report that, under conditions that emulate the physiologically relevant bursting activity of ventral midbrain neurons in vivo, α-Syn facilitates DA release. This effect of α-Syn appears to be exquisitely tuned to inhibit rapid depression during repeated bursts and so contribute to short-term plasticity that may underlie reinforcement and motor learning and action selection. This presynaptic facilitation is particularly salient for neurotransmitter systems such as monoamines that engage in extrasynaptic overflow and rely on intermittent activity bursts (21). Further research may indicate if this form of regulation occurs in other synapses that undergo prolonged bursting, such as those of the torpedo electric organ, neuromuscular junction, motor cortex, and the sympathetic nervous system.

## Materials and Methods

### Mice.

All experimental procedures followed NIH guidelines and were approved by Columbia University’s Institutional Animal Care and Use Committee. Data from male and female mice were combined since there were no differences in the experimental outcome between animal sexes. C57BL/6J (stock no. 000664), background controls (B6129SF2/J, stock no. 101045) and α-Syn knockout (α-SynKO, B6;129X1-Sncatm1Rosl/J, stock no. 003692) were acquired from The Jackson Laboratory (Bar Harbor, ME). The WT mice are a cross between C57BL6/J and B6129SF2/J mouse lines. Synuclein triple knockout (SynTKO) mice were obtained from the laboratory of R.H.E. All experiments were performed on 5- to 8-mo-old mice.

### Surgery.

Mice were anesthetized with isoflurane (SomnoSuite Small Animal Anesthesia System, Kent Scientific, induction 2.5%, maintenance 0.8 to 1.4% in O_2_, 0.35 L/min). A mouse was placed on a circulating warm water heating pad and head-fixed on a stereotaxic frame (Kopf Instruments, Tujunga, CA). Puralube vet ointment was applied on the eye to prevent cornea from drying out. Craniotomy (unilateral, right) was performed using a drill (0.8 mm) to target the specific region of interest with the following coordinates from a mouse brain atlas (58) (mm from Bregma): midbrain, anteroposterior (AP) = −2.9, mediolateral (ML) = +1.0, dorsoventral (DV) = +4; dorsal striatum, AP = +1.2, ML = +1.3, DV = +3.1. Breathing and the depth of anesthesia (toe pinch) were constantly monitored.

### In Vivo Electrophysiology.

Extracellular single-unit recordings of midbrain SN DA neurons were recorded from WT mice under isoflurane anesthesia (1 to 1.2%, unless stated otherwise). We followed a previous procedure (26) where anesthetized mouse was head-fixed on a stereotactic frame with continuous flow of isoflurane (SomnoSuite; Kent Scientific), followed by craniotomy to target midbrain as above (*Surgery*). Glass electrodes (12 to 22 MΩ; Harvard Apparatus) filled with 0.5 M NaCl, 10 mm 4-(2-hydroxyethyl)-1-piperazineethanesulfonic acid (HEPES), 1.5% neurobiotin (Nb) (Vector Laboratories) were used for recording. An Ag/AgCl reference electrode was placed under the skin. Micromanipulator (SM-7; Luigs and Neumann) was used to lower the glass electrodes to the recording site. The spontaneous extracellular single-unit activity of SN DA neurons was recorded for 10 min using an ITC-18 A/D converter (WinWCP software; sampling rate 11 kHz; Heka). The extracellular signals were amplified 1,000× (ELC-03M; NPI Electronics), bandpass-filtered 0.3 to 5 kHz (single-pole, 6 dB/octave, DPA-2FS; NPI Electronics). Midbrain SN dopaminergic neurons were identified by the well-established electrophysiological characteristics: broad biphasic action potential (>1.2 ms), slow firing frequency (1 to 8 Hz), and their firing pattern (regular, irregular, and bursting) (27, 59). For the in vivo data analysis, the mean discharge frequencies were calculated, and interspike interval histograms (ISIHs) (10-ms bins) were plotted for every exported 600-s spike-train. The coefficient of variation (CV)—a measure of irregularity of spiking—was obtained by the ratio of SD to the mean. The pattern of the in vivo spike-train was visually classified using the output from autocorrelation (measures ISIs to the nth order) histogram (ACH, 1-ms bins, smoothed with a Gaussian filter [20 ms]) (26, 60–62) using R statistical computing (https://www.r-project.org). The observed in vivo firing patterns were as follows: regular (pacemaker), irregular, or bursty. The burst within the spike-train was identified according to the 80/160 ms criterion (27). A spike-train was classified as a regular if the ACH had a minimum of three consecutive oscillations with decreasing amplitude. An irregular spike-train had an ACH with a plateau (equal probability of occurrence of specific ISI throughout the spike-train). The bursty pattern had an ACH displaying a narrow initial peak (bursts), with a shallow trough followed by a broader peak (pause). The irregular bursty pattern had an ACH with an initial narrow and steep peak, followed by a plateau. IgorPro 6.02 (Neuromatic; WaveMetrics) software was used for the spike-train analysis.

### Juxtacellular Labeling of Single Neurons In Vivo.

To map the anatomical localization of dopaminergic neurons following extracellular single-unit recordings, neurons were labeled with Nb using a juxtacellular in vivo labeling technique (63). Microiontophoretic current was applied (1 to 10 nA positive current, 200 ms on/off pulse, ELC-03M; EPC-10, as external trigger; NPI Electronics) via the recording electrode, with continuous monitoring of the single-unit activity. The labeling event was considered successful when the firing pattern of the neuron was modulated during current injection (i.e., increased activity during on-pulse and absence of activity in the off-pulse) and the process was stable for a minimum of 25 s, followed by the spontaneous activity of the neuron after modulation. This procedure, in combination with immunohistochemical identification using TH antibodies (see *Histology* section below), resulted in the exact mapping of the recorded DA neuron within the SN subnuclei (58).

### In Vivo FSCV.

Mice were anesthetized as described in the *Surgery* section, and craniotomy was performed to target right midbrain and the striatum (*Surgery*). A 22G bipolar stimulating electrode (P1 Technologies) was lowered to reach ventral midbrain (between 4 and 4.5 mm). The exact depth was adjusted for maximal DA release. An Ag/AgCl reference electrode was placed under the skin via a saline bridge. A carbon fiber electrode (5-μm diameter, cut to ∼150-μm length; Hexcel Corporation) was used to measure the evoked DA release from the dorsal striatum. To verify electrode positions in FSCV experiments, the carbon fiber electrode was briefly dipped into DiI solution (1:1,000; Thermo Fisher) and air dried. This electrode was lowered to the described coordinates. A triangular voltage wave was applied to the electrode every 100 ms (−450 to +800 mV at 294 mV/ms versus Ag/AgCl). Signals were digitized using an ITC-18 board (Instrutech; HEKA) and recorded with IGOR Pro-6.37 software (WaveMetrics). The current measured during the triangular voltage wave was measured with an Axopatch 200B amplifier (Axon Instruments) with a 5-kHz low-pass Bessel Filter setting and sampled at 25 kHz.

The stimulating electrode position was identified using the electrode track in the brain tissue. Constant current (400 μA) was delivered using an Iso-Flex stimulus isolator triggered by a Master-8 pulse generator (AMPI, Jerusalem, Israel). A single-burst stimulation consisted of 30 pulses at 50 Hz (0.6 s). Repeated-burst stimulation consisted of six 30-pulse/50-Hz bursts delivered at 5-s intervals. Electrodes were calibrated using known concentration of DA in artificial cerebrospinal fluid (ACSF). A custom-written procedure in IGOR Pro was used for the data acquisition and analysis (sulzerlab.org/download.html).

### In Vivo Fiber Photometry.

Mice were anesthetized using isoflurane and prepared for surgery and craniotomy targeted to the midbrain (*Surgery*). A Nanoject was used to inject 100 nL of AAV9-Flex-GCamp6f (Addgene #100833) at a rate of 18.4 nL/min, followed by a 5-min wait period before removing the needle. Mice were allowed to recover for 3 wk to provide sufficient GCamp6f expression. On the day of the experiment, mice were anesthetized, and a craniotomy was performed to target midbrain and the striatum (*Surgery*). An optical fiber (0.22 numerical aperture, 300-μm diameter, 3.5-mm length; Doric) was lowered into the dorsal striatum while a stimulating electrode was placed in the ventral midbrain. A Doric photometry system (Doric Lenses Inc.) was used for light excitation and detection. Two LEDs were used, one at wavelength 405 nm for measuring background fluorescence, and a second at 465 nm to excite GCamp6f. The same fiber was used to detect the light emission. Doric Neuroscience Studio software was used to record the data. Calcium traces were analyzed with custom-written codes using IgorPro6.2.

### Ex Vivo Slice Electrophysiology.

Mice were euthanized by cervical dislocation, and coronal 270-µm-thick midbrain slices were prepared on a vibratome (VT1200; Leica, Sloms, Germany) in oxygenated ice cold cutting-ACSF containing 195.2 mM *N*-methyl-d-glucamine (NMDG), 2.5 mM KCl, 30 mM NaHCO_3_, 20 mM Hepes, 10 mM MgCl_2_·6H_2_O, 1.25 mM NaH_2_PO_4_, 25 mM d-glucose, 2 mM thiourea, 5 mM Na^+^-ascorbate, and 3 mM Na^+^-pyruvate (pH 7.4, 290 ± 5 mOsm). Slices were then transferred to oxygenated normal ACSF containing 125.2 mM NaCl, 2.5 mM KCl, 26 mM NaHCO_3_, 1.3 mM MgCl_2_·6H_2_O, 2.4 mM CaCl_2_, 0.3 mM NaH_2_PO_4_, 0.3 mM KH_2_PO_4_, and 10 mM d-glucose (pH 7.4, 290 ± 5 mOsm) at 34 °C and allowed to recover for at least 40 min before the recordings.

Electrophysiological recordings were performed on an upright Olympus BX50WI (Olympus, Tokyo, Japan) microscope equipped with a 40× water immersion objective, differential interference contrast (DIC) optics, and an infrared video camera. All recorded neurons were in the SN anatomical area and identified as dopaminergic by their spontaneous firing frequency (1 to 4 Hz). Slices were transferred to a recording chamber and maintained under perfusion with normal ACSF (2 to 3 mL/min) at 34 °C. Patch pipettes (3 to 5 MΩ) were pulled using P-97 puller (Sutter instruments, Novato, CA) and filled with ACSF. Loose seal (∼40 to 70 MΩ) cell-attached patch clamp recordings were performed in current clamp mode with a MultiClamp 700B amplifier (Molecular Devices, San Jose, CA) and digitized at 10 kHz using an InstruTECH ITC-18 board (HEKA, Holliston, MA). To perfuse isoflurane, the air was mixed with isoflurane (100%) using a commercial vaporizer (SomnoSuite; Kent Scientific). Isoflurane (1 and 3%) and ACSF were bubbled at the same time just before the media was perfused to the slice. After establishing stable baseline spontaneous action potential (sAP) firing in normal ACSF, isoflurane was perfused for 10 min. Data were acquired using WINWCP software (developed by John Dempster, University of Strathclyde) and analyzed using Clampfit (Molecular Devices) and Matlab (MathWorks, Natick, MA).

### Histology and Imaging.

For immunocytochemical detection, mice were transcardially perfused with ice-cold paraformaldehyde (PFA) (4%), and the brains were isolated and stored in PFA overnight. Next, brains were sectioned into 50-µm (for TH and biotin staining) or 100-µm (for DiI visualization) slices using a Leica VT2000 vibratome (Leica, Richmond, VA). Free-floating sections were rinsed with phosphate-buffered saline (PBS) (3 × 10 min) followed by 1-h blocking at room temperature using 10% (normal donkey serum [NDS] in PBS/Triton-X 100 (TX) (0.1 to 0.3%). Sections were then incubated overnight with primary antibody against TH diluted in 2% NDS in PBS/TX (1:1,000, rabbit anti-TH, AB152; Millipore). The sections were rinsed with PBS (3 × 10 min) and incubated for 6 h with secondary donkey anti-rabbit 488 antibodies (1:1,000, A11006; Thermo Fisher Scientific) and streptavidin 594 (1:750, S32356; Invitrogen) diluted in 2% NDS in PBS/TX. The sections were washed with PBS (3 × 10 min), arranged along the caudo-rostral axis, mounted onto Superfrost plus microscope slides (Fisher Scientific), and allowed to air dry. Once the sections were partially attached to the glass, the coverslips were mounted using an antifading mounting medium (Vectashield; Vector Laboratories). The sections were visualized using confocal laser scanning microscopy (CLSM) (TCS SP8, 5×, 10×, and 60× objectives; Leica) operated by Leica LAS X Core software.

### Statistical Analysis.

The data in the figures are represented as mean with SEM. Grouped data (with one or two different conditions) were analyzed using repeated measures one/two-factor within-subject analysis of variance (one-way or two-way ANOVA, mixed effects model), followed by Bonferroni’s multiple comparisons test. The dependent measures were the following: burst numbers and genotypes. Significance was assumed if *P* < 0.05. The values for each analysis (mean + SEM, degrees of freedom [F], and the number of data points) are presented in the text. GraphPad Prism Software (GraphPad Software, San Diego, CA) was used for the data plots and statistical analysis.

## Supplementary Material

Supplementary File

## Data Availability

The source code for our model has been deposited on GitHub at https://github.com/dsulzer/sulzerlab-analysis and at our laboratory website sulzerlab.org/download.html.
