# Supplementary material for: A dual role for α-synuclein in facilitation and depression of dopamine release from substantia nigra neurons in vivo
Source: Proc Natl Acad Sci U S A. 2020 Dec 3;117(51):32701–10. doi: 10.1073/pnas.2013652117 (PMC7768743; doi:10.1073/pnas.2013652117)
Supplement: Supplementary File [file pnas.2013652117.sapp.pdf]

Supplementary Information for

A dual role for α-synuclein in facilitiation and depression of dopamine release from substantia nigra neurons *in vivo*

Mahalakshmi Somayaji[1], Stefano Cataldi[1], Se Joon Choi[1], Robert H. Edwards[2], Eugene V. Mosharov[1], David Sulzer[1]*

David Sulzer
Email: ds43@cumc.columbia.edu

This PDF file includes:

Figures S1 to S4
Table S1

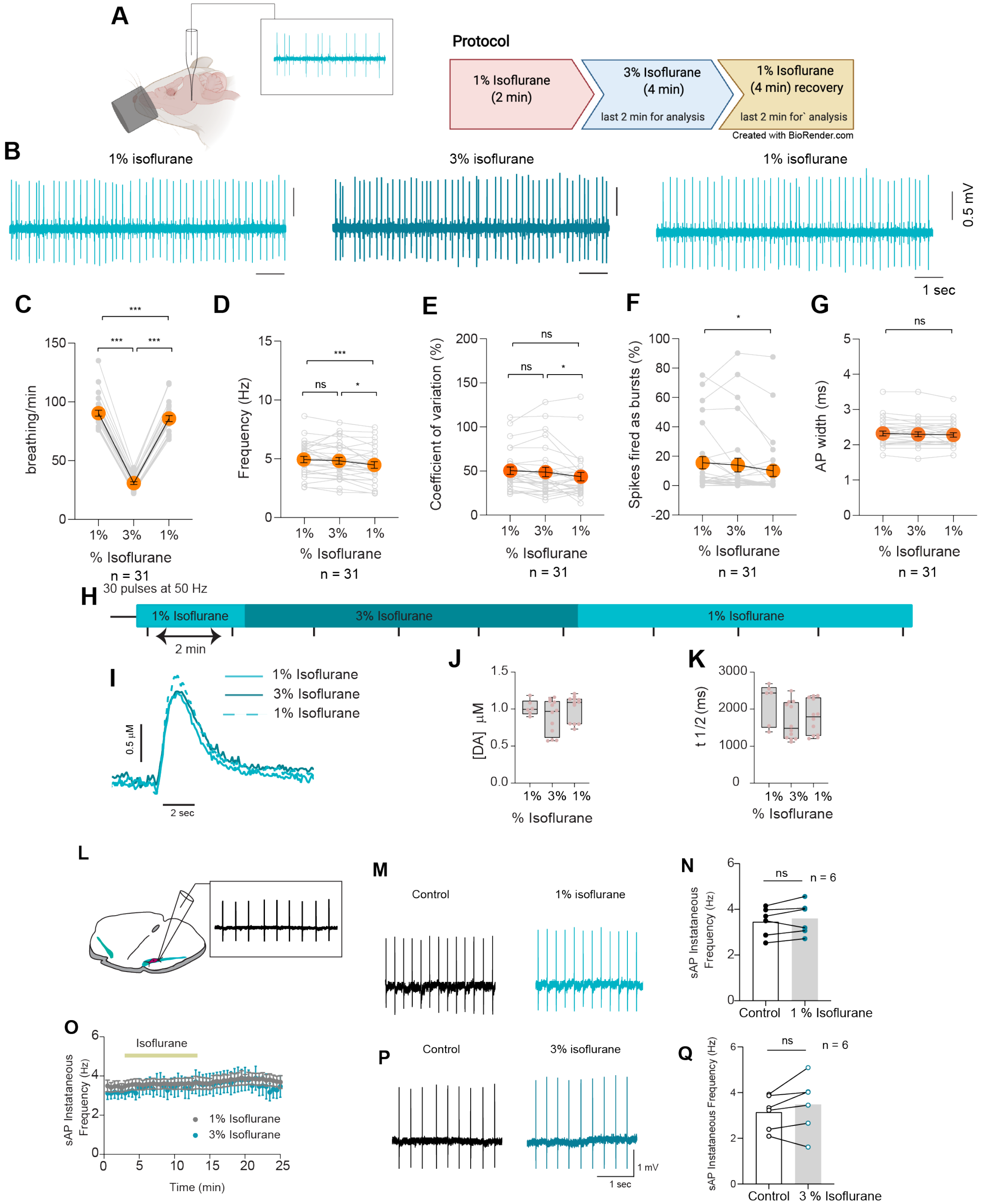

Fig. S1. <u>No difference in firing properties or dopamine release from midbrain dopamine neurons during 1 and 3% isoflurane anesthesia:</u> Recording protocol (*A*) and representative recordings (*B*) of spontaneous activity of putative SN dopaminergic neurons *in vivo* under isoflurane anesthesia. Neuronal activity was recorded for 2 min under 1% isoflurane, followed by an increase of isoflurane to 3% for 4 min and back to 1% for 4 min. Scale bar on B are 0.5 mV and 1 sec. (*C*) Anesthesia-induced changes in the mean breathing rate under 1% (90 ± 3 breathing/min), 3% (31

± 1 breathing/min) and 1% (washout, 86 ± 3 breathing/min) isoflurane. The breathing rate decreases (~65%) with anesthesia depth (*P* <0.0001; 1% vs. 3%, *P* <0.0001; 3% vs. 1%, *P* <0.0001 and 1% vs. 1%, *P* = 0.02 by paired one-way ANOVA). (*D*) Scatterplot showing mean spontaneous firing frequency of DA neurons under 1% (mean ± SEM: 4.9 ± 0.3 Hz), 3% (4.8 ± 0.3 Hz) and 1% (4.5 ± 0.3 Hz) isoflurane anesthesia. (*P* <0.0001; 1% vs. 3%, ns; 3% vs. 1%, *P* = 0.02 and 1% vs. 1%, *P* = 0.0008 by paired one-way ANOVA). (*E*) Mean coefficient of variation (CV) of DA neurons firing under 1% (51 ± 4 %), 3% (49 ± 5 %) and 1% (44 ± 5 %) isoflurane anesthesia (*P* = 0.02; 1% vs. 3%, ns; 3% vs. 1%, ns and 1% vs. 1%, *P* = 0.03). (*F*) Percentage of spikes fired as bursts (SFB) in DA neurons under 1% (15 ± 4 %), 3% (14 ± 5 %) and 1% (10 ± 4 %) isoflurane anesthesia (*P* = 0.03; 1% vs. 3%, ns; 3% vs. 1%, ns and 1% vs. 1%, ns). (*G*) Mean action potential width in neurons under 1% (2.3 ± 0.07 ms), 3% (2.3 ± 0.07 ms) and 1% (2.3 ± 0.06 ms) isoflurane anesthesia (ns). All the scatter plots on C-G depict the mean ± SEM values (red filled circles) and the values in individual neurons (grey circles). Statistical comparison was performed by repeated measure ANOVA with Bonferroni's multiple comparisons test. (*H*) Protocol used for *in vivo* recordings of evoked dopamine release from the dorsal striatum under different concentrations of isoflurane. The cell bodies were stimulated using 30 pulses at 50 Hz every 2 min and the evoked dopamine release was measured under 1% isoflurane, then 3% and back to 1%. (*I*) Representative recordings of the striatal dopamine release under 1% (light teal)-3% (dark teal) -1% (light teal, dotted) isoflurane anesthesia. Scale bar = 0.5 µM/ 2 sec. (*J*) Average evoked dopamine release under 1% (mean ± SEM: 1.0 ± 0.04 µM), 3% (0.9 ± 0.07 µM) and 1% (1.0 ± 0.05 µM) isoflurane anesthesia (*P* = 0.09; 1% vs. 3%, ns; 3% vs. 1%, ns and 1% vs. 1%, ns). (*K*) Boxplot showing the t1/2 of dopamine reuptake under 1% (mean ± SEM: 2173 ± 228 ms), 3% (1675 ± 145 ms) and 1% (1776 ± 135 ms) isoflurane anesthesia (*P* = 0.36; 1% vs. 3%, ns; 3% vs. 1%, ns and 1% vs. 1%, ns). Statistics (J and K): Mixed-Effects analysis with Bonferroni's multiple comparisons test. (*L*) Ex vivo slice electrophysiology of midbrain SN DA neurons. (*M and P*) Representative cell-attached recording of action potentials (sAP) before and after 1 % (M) and 3 % (P) isoflurane application. (*O*) Changes in the sAP in SN dopamine neurons during isoflurane perfusion in the extracellular media. (*N and Q*) The average sAP frequency 3 min before and 3 min after isoflurane perfusion. Individual neurons are shown as a scatter plot while mean values are represented as bar graphs (N: control, 3.5 ± 0.3; 1% isoflurane, 3.6 ± 0.3; Q: control, 3.1 ± 0.3; 3% isoflurane, 3.4 ± 0.5; not-significant by paired t-test; n=6 cells in each group).

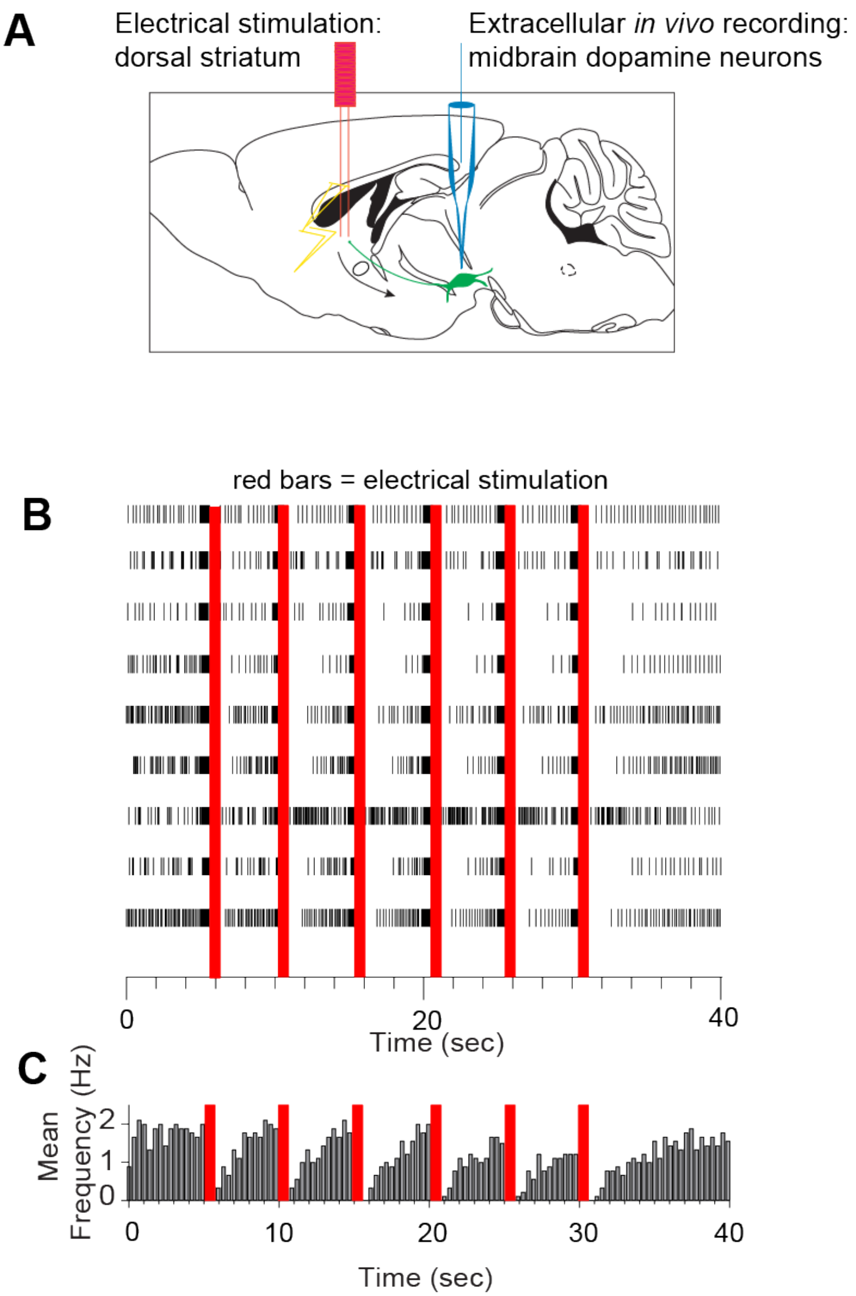

Fig. S2. Antidromic stimulation of midbrain substantia nigra dopamine neurons: **(*A*)** Schematic showing the antidromic stimulation experimental set-up. The dopamine axons within the dorsal striatum were electrically stimulated using repeated burst protocol where a burst of 30 pulses at 50 Hz was applied 6 times with inter-stimulus interval of 5 sec. **(*B*)** Raster plots of spontaneous firing frequency of putative SN dopaminergic neurons in response to the antidromic stimulation (each row represents a single neuron, n = 9). **(*C*)** Histogram showing average AP frequency in the cell bodies of SN neurons. Every striatal electrical stimulation induces a prominent pause in the firing of DA neurons.

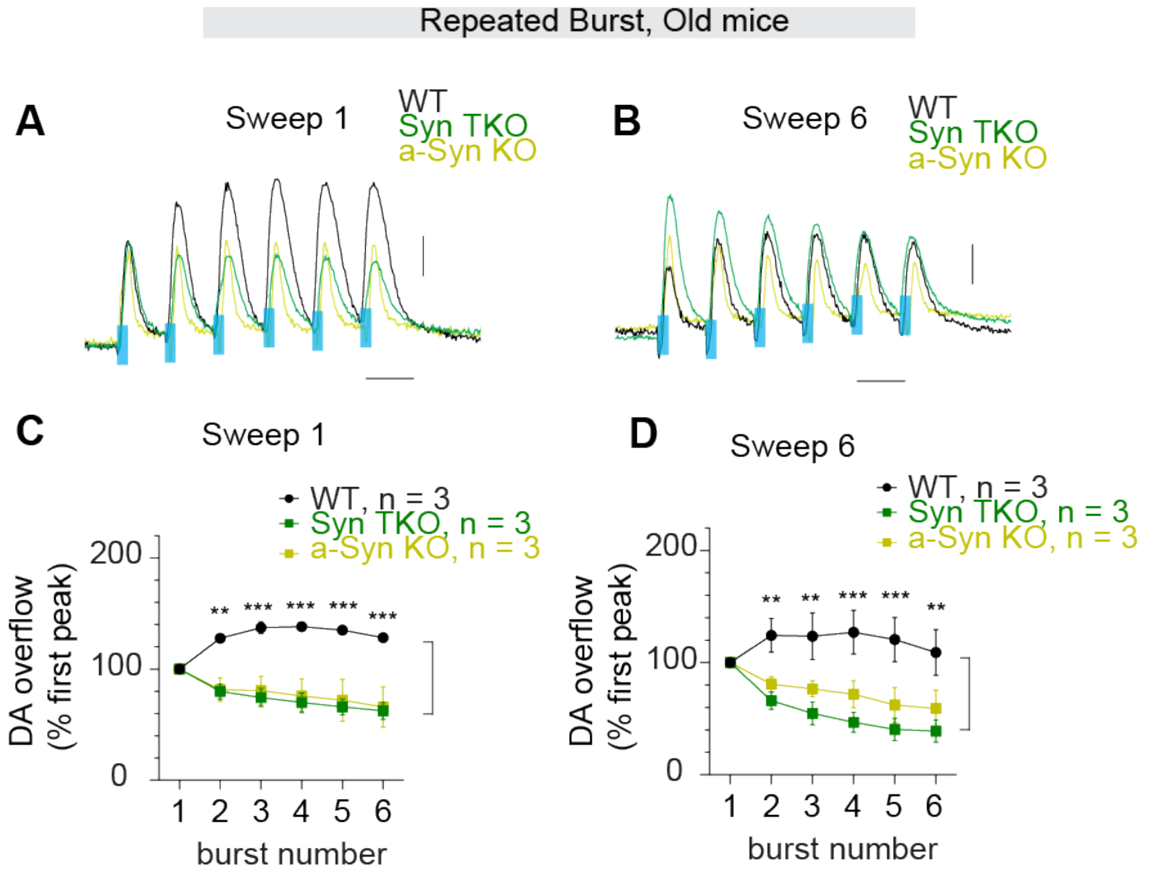

Fig. S3. Synuclein-dependent facilitation of dopamine release during short-interval (5 sec) burst stimuli at older age. **(*A* and *B*)** Representative recordings of evoked dopamine release in sweep 1 (A) and sweep 6 (B) of repeated burst stimulation protocol (Fig 4A) in 10-13-month-old mice (average age: WT = 13 mo; Syn TKO = 13 mo; α-Syn KO = 10 mo). Scalebars are 500 nM and 5 sec. **(*C*)** Facilitation of dopamine release during *sweep 1* in old WT (one-way ANOVA within genotype: $F_{2,10} = 170$, $p = 0.0001$; Bonferonni's multiple comparison: burst 1 vs 2:6, P = 0.003, 0.0002,0.0002, 0.0004, 0.002 ; n = 3) and depression of dopamine release in SynTKO (one-way ANOVA within genotype: $F_{2,10} = 9.7$, $p = 0.005$; Bonferonni's multiple comparison: burst 1 vs 4:6, P = 0.03, 0.01, 0.006; n = 3) and α-SynKO (one-way ANOVA within genotype: $F_{2,10} = 415$, p =0.009 ; Bonferonni's multiple comparison: burst 1 vs 5:6, P = 0.04, 0.006; n = 3) animals. Two-way ANOVA between genotypes: $F_{2,36} = 58$, p = <0.0001; Bonferroni's multiple comparisons test: WT vs Syn TKO, burst 2:6, P = 0.003, 0.0001, <0.0001, <0.0001, <0.0001. WT vs α-Syn KO, burst 2:6, P = 0.004, 0.0004, 0.0001, <0.0001, 0.0001). **(*D*)** Dopamine release during *sweep 6* in old WT (one-way ANOVA within genotype: $F_{2,10} = 18.1$, $p = 0.3$; Bonferonni's multiple comparison: ns; n = 3) and depression of dopamine release in the SynTKO (one-way ANOVA within genotype: $F_{2,10} = 166$, $p < 0.0001$; Bonferonni's multiple comparison: burst 1 vs 2:6, P = 0.003, 0.0003, <0.0001, <0.0001, <0.0001; n = 3) and α-SynKO (one-way ANOVA within genotype: $F_{2,10} = 77.8$, $p = 0.007$; Bonferonni's multiple comparison: burst 1 vs 5:6, P = 0.02, 0.002; n = 3) animals. Two-way ANOVA between genotypes: $F_{2,36} = 33.3$, p = <0.0001; Bonferroni's multiple comparisons test: WT vs Syn TKO, burst 2:6, P = 0.01, 0.002, 0.0003, 0.0003, 0.002. WT vs α-Syn KO, burst 3:6, P = 0.04, 0.01, 0.009, 0.03).

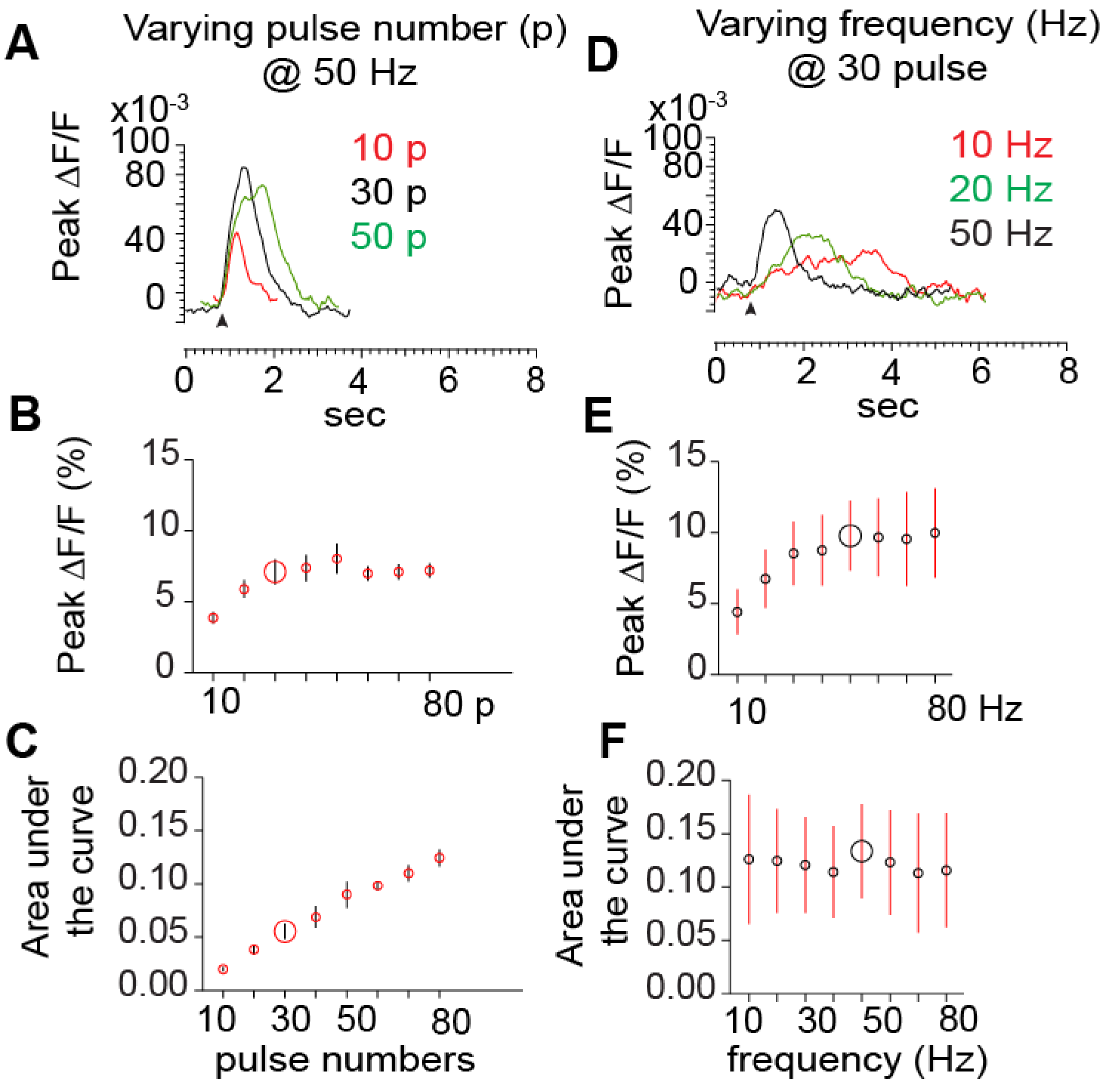

Fig. S4. Analysis of in vivo GCaMP6f response to electrical stimulation. **(*A*)** Evoked calcium response to electrical stimulation at constant frequency (50 Hz) and varying pulse number (10 pulse – red; 30 pulse – black; 50 pulse – green) under isoflurane anesthesia. Arrowhead indicates the initiation of electrical stimulation. **(*B*)** Peak GCaMP6f signal (ΔF/F) at varying pulse number (10-80 p) at a constant stimulation frequency. Note the plateau in the signal peak height from 30 p. **(*C*)** Area under the curve (AUC) measurements for the evoked calcium signals at different pulse number (10-80 p). Note the linear increase in the AUC (despite the lack of change in peak height). **(*D*)** Evoked GCaMP6f response to electrical stimulation at constant pulse number (30 pulses) at a range of stimulus frequencies (10 Hz – red; 20 Hz – green; 50 Hz – black). Arrowhead indicate the initiation of electrical stimulation. **(*E*)** Peak calcium signal (ΔF/F) at varying stimulation frequency (10-80 Hz) at a constant pulse number. Note the plateau in the GCaMP6f signal from 50 Hz. **(*F*)** Area under the curve (AUC) measurements for the evoked calcium signals at different stimulation frequencies (10-80 Hz). Note that change in stimulation frequency has no effect on the AUC. Enlarged circles represent the chosen stimulation parameters. Error bars represent SEM (n= 4 mice).

Table S1. Comparison of *in vivo* firing properties of dopamine neurons from this manuscript (first highlighted row) with data from other publications.

Table S1: *In vivo* firing properties of Dopamine neurons in anesthetized animals:

Reference	Species	Anesthesia	Mean firing Frequency (Hz) ± SEM	Coefficient of Variation (%) ± SEM	Mean Burst Frequency (Hz) ±SEM	SFB (%) ± SEM
**This manuscript**	**Mice**	**Isoflurane (1-3%)**	**4.9 ± 0.3**	**51 ± 4**	**29.1 ± 5**	**15.4 ± 4**
Farassat et. al., (2019)	Mice	Isoflurane (1.2%)	3.51 ± 0.4	62.92 ± 8	21.4 ± 6	11.93 ± 4.2
Subramaniam et.al., (2014)	Mice	Isoflurane (0.9-1.4%)	3:4 ± 0:3	58 ± 5	13.7 ± 0.3	12.0 ± 5
Branch et. al., (2013)	Mice	Urethane (1.8 g/Kg i.p)	3.1 ± 0.6	na	na	1.9 ± 0.6
Schiemann et. al., (2012)	Mice	Isoflurane (1-1.4%)	4.2 ± 0:3	74 ± 6	14 ± 0.3	22 ± 5
Jeong et. al., (2012)	Rats	Chloral hydrate (400 mg/kg, i.p.)	4.3 ± na	na	na	22.7 ± na
Jeong et. al., (2012)	Rats	Halothane (1%)	7.2 ± na	na	na	43.6 ± na
Herrik et. al., (2010)	Mice	Urethane (1.8 g/kg i.p)	5.2 ± 1.3	26.3 ± na	na	1.0 ± na
Bishop et. al., (2010)	Rats	Urethane (2.0 g/kg ip)+ ketamine (20 mg/kg ip)	3.8 ± 1.5	69 ± 16	na	11.7 ± 8.7
Brown et. al., (2009)	Rats	Urethane (1.3 g/kg, i.p.); suppl. doses of ketamine (30 mg/kg, i.p) and xylazine (3 mg/kg, i.p.)	3.6 ± 0.3	20 ± 2	na	26.9 ± na
Ishida et. al., (2009)	Rats	Urethane ((1.2g/kg i.p)	3.6 ± 0.2	40.4 ± 4.7	na	na
Chen et. al., (2009)	Rats	Chloral hydrate (300 mg/kg, i.p.)	3.7 ± 0.2	46 ± 3.0	na	41.6 ± 5
Zhang et. al., (2008)	Rats	Chloral hydrate (400 mg/kg, i.p.)	3.4 ± 0.1	40.5 ± 1.4		4.2 ± 0.7
Fà et. al., (2003)	Rats	Chloral hydrate (400 mg/kg, i.p.)	3.5 ± 0.4	na	13.7 ± 2.4	13.2 ± 2.9
Paladini et. al., (2003)	Mice	10 ml/kg of Ketamine (2.5%)-xylazine (1%)-acepromazine (0.5%)	3.6 ± 1.3	65 ± 8	na	34 ± na
Tepper et. al., (1995)	Rats	Urethane (1.3 g/kg, i.p.)	3.8 ± 0.2	na	na	4.5 ± 1.0
Clark & Chiodo (1988)	Rats	Chloral hydrate (400 mg/kg, i.p.)	3.8 ± 0.2	na	na	8.2 ± 1.5
Grace & Bunney (1984,b)	Rats	Chloral hydrate (400 mg/kg, i.p.)	4.5 ± 1.7	na	na	29.0 ± 24.0

***In vivo* firing properties of Dopamine neurons in awake animals:**

Reference	Species		Mean firing Frequency (Hz) ± SEM	Coefficient of Variation (%) ± SEM	Mean Burst Frequency (Hz) ± SEM	SFB (%) ± SEM
Duvarci et. al., (2019)	Mice	Awake	3.6 ± 0.2	na	na	na
Dodson et. al., (2016)	Mice	Awake head-fixed	5.8 ± na	50 ± na	na	na
Zaghloul et. al., (2009)	Humans	Awake	3.5 ± 0.4	155 ± 15.6	20.2 ± 0.6	57.9 ± 3.1
Bayer et. al., (2007)	Monkeys	Awake	5.3 ± 1.5	61 ± 16	25-30 ± na	na
Robinson et. al., (2004)	Mice	Awake	4.6 ± 0.4	na	40.2 ± 2.6	48.9 ± 5.7
Fà et. al., (2003)	Rats	Awake	3.6 ± 0.4	na	na	23.5 ± 2.7
Hyland et. al., (2002)	Rats	Awake	3.7 ± 1.5	60.8 ± 23	20.1 ± 4.6	20.9 ± 21
Freeman et. al., (1985)	Rats	Awake	5.5 ± 1.4	na	15.2 ± 3.9	46 ± 23
Steinfels et. al., (1981)	Cats	Awake	3.4 ± 0.3	na	na	na
